# Optimization of Ionic Liquid-Assisted Extraction of Biflavonoids from *Selaginella doederleinii* and Evaluation of Its Antioxidant and Antitumor Activity

**DOI:** 10.3390/molecules22040586

**Published:** 2017-04-07

**Authors:** Dan Li, Yan Qian, Yu-Jia Tian, Shi-Meng Yuan, Wei Wei, Gang Wang

**Affiliations:** School of Pharmacy, Zunyi Medical College, Zunyi, Guizhou 563003, China; 13984515977@163.com (D.L.); 18785413312@163.com (Y.Q.); 18785413632@163.com (Y.-J.T.); 18311542078@163.com (S.-M.Y.); 18076271737@163.com (W.W.)

**Keywords:** *Selaginella doederleinii* Hieron, flavonoid, antioxidant, anticancer, ionic liquids

## Abstract

As new green solvents, ionic liquids (ILs) have been generally applied in the extraction and separation of natural product. In this study, microwave assisted extraction based on IL (IL-MAE) was firstly employed to extract total biflavonoids from *Selaginella doederleinii*. Based on single-factor experiment, microwave power (300–700 W), extract time (30–50 min) and extract temperature (40–60 °C) on total bioflavonoids and antioxidant activities of the extracts were further investigated by a Box-Behnken design of response surface methodology (RSM) selecting total bioflavonoids yields and IC_50_ of radical scavenging as index. Besides antioxidant activity of the extract was evaluated by a 2,2-diphenyl-1-picrylhydarzyl (DPPH) and 2,2′-azinobis-(3-ethylbenzthiazoline-6-sulphonate (ABTS) radical scavenging assay, ferric reducing power assay and chelation of ferrous ions assay, and then anticaner activity was also researched against A549 cell line and 7721 cell line. The results illustrated that three factors and their interactions could be well suited for second-order polynomial models (*p* < 0.05). Through process parameters, optimization of the extract (460 W, 40 min, and 45 °C) and detection of bioactivity, the yield of total bioflavonoids was 16.83 mg/g and IC_50_ value was 56.24 μg/mL, respectively, indicating the extract has better anti-oxidation effect and antitumor activity. Furthermore, IL-MAE was the most efficient extracting method compared with MAE and Soxhlet extraction, which could improve extraction efficiency in a shorter time and at a lower temperature. In general, ILs-MAE was first adopted to establish a novel and green extraction process on the yields of total biflavonoids from *S. doederleinii*. In addition, the extract of containing biflavones showed potent antioxidant and anticancer capacity as a utilized valuable bioactive source for natural medicine.

## 1. Introduction

As a traditional Chinese medicine, *Selaginella doederleinii* Hieron is a well-known perennial pteridophyte plant growing in South and Southwestern of China. It has the functions of eliminating wind, scattering frigidity, detumescence, and treating cough [1]. The herb was commonly adopted to treat some major diseases in clinic, for example chorionic carcinoma, malignant hydatidiform mole, nasopharyngeal carcinoma, esophageal cancer, gastric cancer, liver cancer, lung cancer, cervical cancer, and so on [2]. In the reported research, different types of compounds have been discovered from *S. doederleinii*, including biflavonoid, alkaloid, xylogen, sterol, and organic acid [3,4,5,6,7]. 

**Figure 1:**
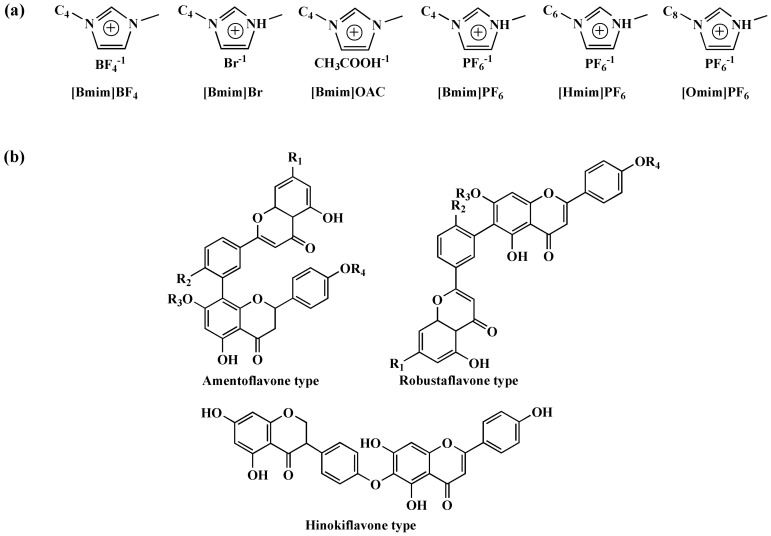
Chemical structures for ILs (**a**); Chemical structures of bioflavonoids in *Selaginella doederleinii* (**b**).

The content of biflavonoids of *S. doederleinii* (just like apigenin and its methoxyl/hydroxyl substituents) is very high and its composition is complex and diverse. Since Lin and colleagues [8] first separated four compounds of biflavone in 1994 from *S. doederleinii,* scholars have separated near 20 biflavonoids. The pure biflavonoid compounds were divided into the three types, which are amentoflavone-type(C3′-C8′′), robustaflavone-type(C3′-C6′′), and hinokiflavone-type(C4′-O-C6′′) biflavonoids, respectively (see Figure 1b). Most bioflavonoids have prominent antioxidant characteristics [9] and show a wide range of biological activity, such as cancer chemopreventive properties, anti-inflammatory, anti-microorganism, and antioxidative effects [10,11,12,13,14].

Anti-tumor effect is an important pharmacological activity of *S. doederleinii*. Acording to Kosμge reported [15], the ethanol extract in *S. doederleinii* could inhibite the growth of ehrlich ascites tumor of mice in vivo and vitro experiments. Lee [16] found that the methanol extracts from *S. doederleinii* showed strong inhibition effects against colon cancer cells, bronchoalveolar cancer, and chronic myeloid leukemia in vitro. Moreover, anti-tumor activity of the methanol extracts was far higher than that of ellipticine as a positive reference.

On the other hand, it has been proven by modern medical research [17,18] that reactive oxygen free radical is associated with some diseases including senescence, malignant tumor, atherosclerosis, diabetes, senile dementia, and parkinsonism [19]. When free radical metabolism is in disorder, the antioxidant can be supplied to the body to improve its conditions. However, it was reported that chemosynthetic antioxidants such as BHT and BHA could inhibit the respiratory enzyme activity in human body and excessive use may result in teratogenesis and cancer. Therefore, it has been a hot topic to search for natural antioxidant from various plants [20]. Some research showed that volatile oils, flavonoids, and alkaloids all can be used as potential antioxidants [21]. However, related activity for *S. doederleinii* has not been focused on research.

Ionic liquids (ILs) are usually deemed as green solvents because of their polarity, viscosity, hydrophobicity, and selectivity [22], which have been widely used in the extraction and separation of natural product. They are also known as ‘designer solvents’ and can be designed by various coupling of different anions and cations [23], and their application is successful for extraction of flavonoids [24], phenolic acids [25], alkaloids [26], terpenoids [27], and other compounds [28] from natural resource. During the last few years, the extraction methods of ILs based-microwave assisted extraction (ILs-MAE) have been developed and they were found superior in extraction efficiency, economic expense, and so on [29]. For instance, Du et al. [30,31] extracted trans-resveratrol from *Rhizoma Polygoni Cuspidati* and lycorine, galanthamine and lycoramine from Lycoris with 1-alkyl-3-methylimidazole ILs through microwave-assisted extraction (MAE). The experimental results showed that the extraction method can not only increase the yields of several active ingredients, but also improve the extraction efficiency; it can replace organic reagents as a kind of greener extractant. Compared with ethanol refluxing technique, the method can obtain higher yields in shorter extraction time.

Therefore, the general objective of this study was to develop and optimize the IL-MAE process on the yields of biflavonoids and IC_50_ of 2,2-diphenyl-1-picrylhydrazyl (DPPH) radicals scavenging from *S. doederleinii* with Box-Behnken design (BBD). The extraction conditions were first studied by traditional single-factor experiment followed the optimization with response surface methodology (RSM) and correlation analysis was evaluated between biflavonoids content and antioxidant activity under the optimal conditions. In addition, antioxidant and anti-tumor activities of the extract were analyzed in order to evaluate its potential as a natural drugs supplement. 

## 2. Results and Discussion

### 2.1. Screening Different ILs

**Figure 2:**
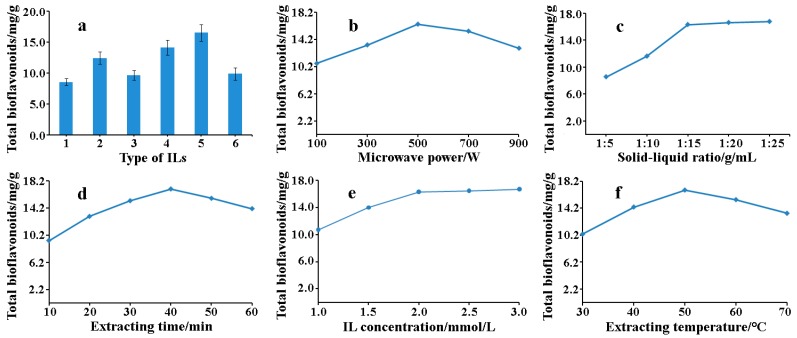
Effect of types of ILs (**a**). 1. (Bmim) (OAC), 2. (Bmim) (BF_4_), 3. (Bmim) (Br), 4. (Bmim) (PF_6_), 5. (Hmim) (PF_6_), 6. (Omim) (PF_6_). Extraction parameters were as follows: 10 g *S. doederleinii*, IL concentration of 2.0 mmol/L, ratio of solution to raw of 1:10 g:mL, microwave power of 500 W and extraction time of 10 min. Effects of extraction parameters on total biflavones yields of *S. doederleinii* extracts: (**b**) Effect of microwave power on total biflavones yields; (**c**) Effect of solid-liquid ratio on total biflavones yields; (**d**) Effect of extraction time on total biflavones yields; (**e**) Effect of ILs concentration on total biflavones yields; (**f**) Effect of extraction temperature on total biflavones yields.

The structure of ILs has significant influence on extraction of flavonoids from natural products [32], which might affect the extraction yields of biflavonoids. Six kinds of ILs(see Figure 1a) ethanol solution were chosen to compare different structure on the influence of extraction yields, which had different carbon chain lengths (C-4–C-8) in the cation and four anions (PF_6_^−^, OAC^−^, BF_4_^−^, Br^−^). As shown in Figure 2a, (Bmim) (PF_6_) compared with three other ILs based on the same cation, had better extraction effects on account of H-bond formation and hydrophobic effect between PF_6_^−^ and biflavonoids. Furthermore, the alkyl chain length of cation can also decide ILs’ extraction efficiency towards target components. (Hmim) (PF_6_) had prominent extraction efficiency towards target products among these six ILs with increasing alkyl chain length in that increase of lipophilicity and hydrophobic effect. However, when carbon chain lengths were C-8, the yields of biflavonoids based (Omim) (PF_6_) obviously declined. It might be that stereo-hindrance effect enhanced between IL and biflavonoids and hydrophobic effect decreased. Thus, our results showed that (Hmim) (PF_6_) was selected as the optimal IL.

### 2.2. Single Factor Experiment

In the preliminary study, the influence of several factors (microwave power, solid-liquid ratio, extracting time, ionic liquid concentration, extraction temperature) on total biflavonoids of the extract from *S. doederleinii* were studied and evaluated.

#### 2.2.1. Effect of Microwave Power

Optimization of the microwave power was essential in MAE and the single-factor experiments were carried out at 100, 300, 500, 700, and 900 W, respectively. Other extraction conditions were set as follows: IL concentration, 2.0 mmol/L; solvent-material, 1:10 g:mL; irradiation time, 10 min; temperature, 50 °C. Figure 2b indicated the effect of extraction process on the yields of biflavnoids with variation of microwave power. When microwave power increased from 100 W to 500 W, it showed that the yield of biflavonoids changes from 10.71 mg/g to 16.44 mg/g. Nevertheless, the extraction yield of biflavnoids obviously decreased with further increases of microwave power from 500 W to 900 W. That extraction yields at 700 W and 900 W were 15.42 mg/g and 12.93 mg/g, respectively. As regards the extraction yield decline, it could be the thermal degradation of biflavonoids under the conditions of higher microwave power. The heat generated by increasing microwave power in the plant cells, may be too strong to destroy the phytochemicals that were not recovered at higher power levels. The results illuminated that the extraction process at higher microwave power levels did not insure better extract yields than those extracted at medium power. According to the report of Ahmad and Langrish [33], there are also similar results that the amounts of total phenolic acids decreased with increased microwave power (900 W). Therefore, three levels of power including 300, 500, and 700 W were used in the subsequent optimization study of RSM.

#### 2.2.2. Effect of Solid-Liquid Ratio

Different solid to liquid ratios (1:5, 1:10, 1:15, 1:20, and 1:25 g:mL) were employed for the effect of different solid-liquid ratios on total biflavonoids of the extract of *S. doederleinii*, and the other extraction conditions were set as follows: ILs concertration, 2.0 mmol/L; microwave power, 500 W; irradiation time, 10 min; temperature, 50 °C. As shown in Figure 2c, the yields of biflavnoids were increasing from 8.55 mg/g to 16.29 mg/g with improvement of the solid-liquid ratio changing from 1:5 to 1:15. However, when the solid-liquid ratio was in the range of 1:15–1:30, the product yield had little change. The reason on the increased yield was that higher solid-liquid ratio could accelerate mass transfer in extraction process and promote the diffusion of more biflavones into the solvent medium [34,35]. Therefore, after considering the difficulties of post-processing and the waste solvent, 1:15 was selected as the optimal solid-liquid ratio. 

#### 2.2.3. Effect of Extraction Time

The extraction process on total biflavonoids of the extracts was carried out at 10, 20, 30, 40 min, 50, and 60 min, respectively and the results were exhibited in Figure 2d. Other extraction conditions were set as follows: according to solid-liquid ratio (l:10 g:mL) by adding ILs solution (2.0 mmol/L), extraction power (500 W), extract time (10 min), extraction temperature (50 °C). When began to heat the sample in the microwave, the product content was increasing until extraction time for 40 min. Then the biflavones of the extract were reducing from 40 min to 60 min and the maximum content of biflavone was 17.01 mg/g at 40 min. It was concluded that microwave irradiation might promote the diffusion of target compounds from material to solvent and accelerate the establishment for dissolution within a very short time. However, the structure of biflavnoids might be degraded after a long exposure to microwave irradiation. Thus, microwave powers of 30, 40, and 50 min were investigated in the next optimization study of RSM.

#### 2.2.4. Effect of ILs Concentration

The effects of different ILs concentration (1.0, 1.5, 2.0, 2.5, 3.0 mmol/L) on total biflavones of the extracts were compared each other, and the results were shown in Figure 2e. With improvement of ILs concentration changing from 1.0 mmol/L to 2.0 mmol/L, the yield of biflavnoids was increasing from 10.71 mg/g to 16.32 mg/g. However, when IL concentration was 2.0–3.0 mmol/L, the product yield had no obvious change. In the reaction, ILs could effectively absorb microwave energy in the microwave field, which made the system temperature rise rapidly, and was dissolved with ethyl alcohol each other caused most of dissolution heat generated, which was better able to dissolve the solute. In addition, between ILs and TBE, a series of mutual forces were formed including polar bond, hydrogen bond, and п-п etc., which promote extraction efficiency of biflavonoids. Thus, after considering the experiment cost and environmental protection, 2.0 mmol/L was selected as the optimal IL concentration. 

#### 2.2.5. Effect of Extracting Temperature

To examine the effect of extraction temperature on extraction efficiency, the results at different extraction temperature (30, 40, 50, 60, and 70 °C, respectively) was investigated. Other extraction conditions were set as follows: solid-liquid ratio (l:10 g:mL) by adding ILs solution (2.0 mmol/L), extraction power (500 W), extract time (10 min). Figure 2f listed the effect of extraction temperature on the yields of biflavones. When extraction temperature was rising from 30 °C to 50 °C, it showed that biflavnoids content was clearly increased changing from 10.38 mg/g to 16.86 mg/g. However, the extraction yield of biflavnoids obviously decreased with further increases of extraction temperature from 50 °C to 70 °C. That extraction yields at 60 °C and 70 °C were 15.45 mg/g and 13.44 mg/g, respectively. The results indicated that biflavones from *S. doederleinii* reached a balance of desorption and solubility at 50 °C, and could be oxidized at higher temperature because of thermally unstable property [36,37]. Therefore, extraction temperature of 40, 50, and 60 °C were used in the following optimization study of RSM.

### 2.3. Analysis of Response Surfaces

#### 2.3.1. Fitting the Model

Response surface optimization is superior to traditional single factor optimization on account of the interactions evaluation of different variables. To further investigate the relationship among the factors in ILs-MAE, several independent variables (extraction temperature, extraction time, and solid to liquid ratio) were optimized by BBD of RSM based on the preliminary single factor test. Table 1 and Table 2 exhibited the predicted quadratic model based on analysis of variance (ANOVA). The results indicated that the proposed models were significant with *p*-value < 0.0001 and 0.0001, the coefficient of determination (*R*^2^) of TBE and IC_50_ were 0.9862 and 0.9879, and the adjusted coefficients of determination (*Adj. R*^2^) of TBE and IC_50_ were 0.9753 and 0.8756, respectively. In addition, the lack of fit of total biflavone and IC_50_ was not significant (*p* > 0.05). Therefore, the models were suitable to predict the variation of total biflavonoid yield and antioxidant activity accurately.

Equation (1) showed that enhancing microwave power (X_1_), extraction time (X_2_) and extraction temperature (X_3_) the biflavonoid content could clearly increase biflavonoid yields (Y_1_). Besides the interactive mode X_2_X_3_ had a negative correlation effect, X_1_X_2_ and X_1_X_3_ had a beneficial effect on the yields of total biflavonoids (Y_1_). Equation (2) illustrated that by means of addition of microwave power (X_1_), extraction time (X_2_), and extraction temperature (X_3_) IC_50_ (Y_2_) can markedly reduce. Moreover, X_2_X_3_ possessed a synergistic effect on antioxygenation, while X_1_X_2_ and X_2_X_3_ possessed an inactive effect.

**Figure 3:**
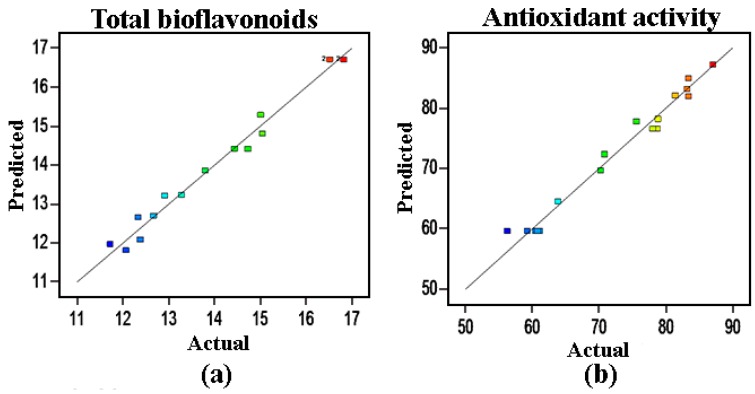
Correlation graph between the predicted and experimental yield values. (**a**) The correlation graph of total biflavonoids content, color points from red to blue indicate values of bioflavonoids changing in the scope of 11 ~ 17 mg/g; (**b**) The correlation graph of antioxidant activity, color points from red to blue indicate values of IC_50_ changing in the scope of 50 ~ 90 μg/mL.

Figure 3 implied that there was a remarkable correlation between the actual values and predicted values. As illustrated by each fact point, the good fit of both models was closely linked to the corresponding regression line. Through the further analysis of RSM on the basis of Equations (1) and (2), the optimal extraction conditions were 460 W, 40 min, and 45 °C with a maximum yield of 16.83 mg/g total biflavonoids and the minimum IC_50_ value of 56.24 μg/mL.

The response surface Equations (1) and (2) are obtained to predict Y_1_ and Y_2_, respectively:

Y_1_ = −50.37+ 0.030X_1_ + 1.12X_2_ + 1.65X_3_ + 0.0000037 X_1_X_2_ + 0.00022 X_1_X_3_ − 0.00072X_2_X_3_ − 0.000044X_1_^2^ − 0.014X_2_^2^ − 0.018X_3_^2^ (1)

Y_2_ = 335.18 − 0.060X_1_ − 8.19X_2_ − 4.42X_3_ − 0.0020 X_1_X_2_ − 0.0023X_1_X_3_ + 0.0065X_2_X_3_ + 0.00026X_1_^2^ + 0.11X_2_^2^ + 0.059X_3_^2^ (2)

#### 2.3.2. Effect of Extraction Parameters

**Figure 4:**
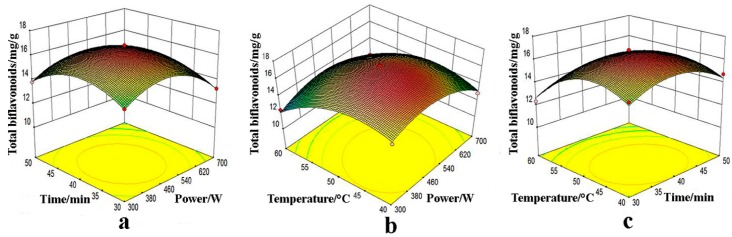
Interaction effects of microwave power and extraction time (**a**); microwave power and extraction temperature (**b**); extraction temperature and time (**c**) on total biflavone yields.

**Figure 5:**
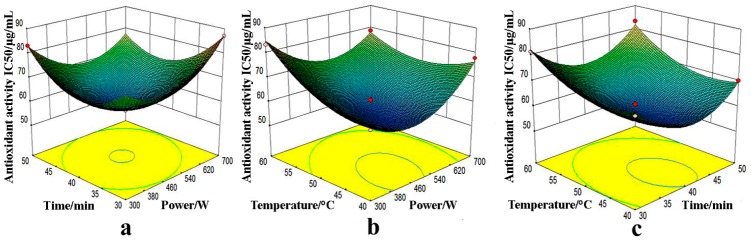
Interaction effects of microwave power and extraction time (**a**); microwave power and extraction temperature (**b**); extraction temperature and time (**c**) on antioxidant activities.

The interaction effect was exhibited from 3D response surface plots between any two variables when the other one is kept at an immobile optimum level. The relationships between two dependent variables (total biflavonoids content and IC_50_ value) and three factors (microwave power, extraction time, and temperature) were illustrated in Figure 4 and Figure 5.

To evaluate the interaction effect between microwave power and extraction time (X_1_X_2_), the results were investigated in Figure 4a and Figure 5a, in the meantime extraction temperature (X_3_) was kept at an intermediate value 50 °C. When increasing microwave power from 300 W to 500 W and an extraction time from 30 min to 40 min, higher total biflavonoids yield (>16.0 mg/g) was obtained. Nevertheless, after further raising microwave power and extraction time, the output of biflavone was certainly decreased. In addition, as regards DPPH radical-scavenging activity of the extracts, a lower value of IC_50_ (<60.00 mg/mL) occurred at microwave power below 540 W and extraction time above 35 min. As shown in Figure 4a, the interaction of two dependent variables, namely extraction temperature and microwave power, had a notable synergistic effect (*p* < 0.05).

The reciprocity effects of microwave power and extraction temperature on each dependent variable were illustrated in Figure 4b and Figure 5b. Total biflavonoids content (>13.0 mg/g) were clearly increased with the improvement of microwave power from 380 W and 540 W and the temperature from 40 °C to 50 °C. However, the extraction yields were gradually decreased when increasing microwave power and extraction temperature. Lower IC_50_ values (<60.00 μg/mL) of extracts were obtained when the temperature was more than 40 °C and microwave power was from 300 W to 500 W. The interaction between microwave power and extraction temperature was significant (*p* < 0.05) for both models.

To evaluate the interaction effect between extraction temperature and extraction time (X_1_X_2_), the results were investigated in Figure 4c and Figure 5c. At higher temperature (>40 °C) and extraction time (>30 min), total biflavonoid content was obviously increased. At more than 50 °C and over 40 min, as described above, the yield decreased seeing that biflavonoids contained phenolic hydroxyl groups and were susceptible to oxidation. Moreover, regarding the DPPH radical-scavenging activity of the extracts, smaller values of IC_50_ (<60.00 μg/mL) were attained when temperature over 40 °C and extraction time less than 45 min. However, the reciprocity had negative effect on both dependent variables estimated from Figure 4c and Figure 5c (*p* > 0.05).

#### 2.3.3. The Relationship between Total Biflavonoids Content and IC_50_ Value

**Figure 6:**
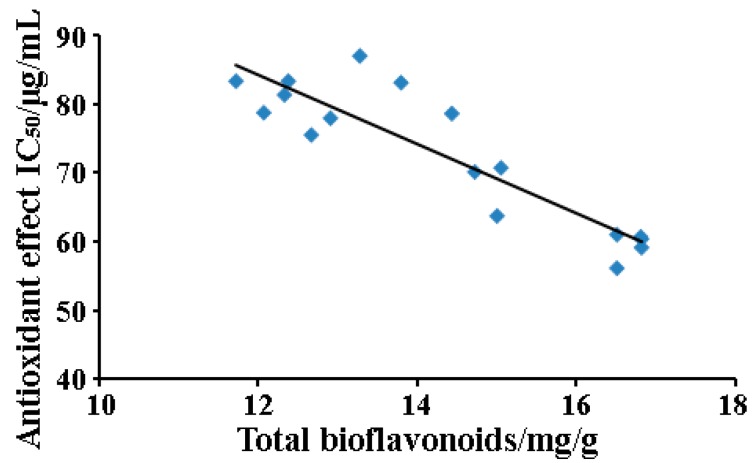
The correlation between total biflavonoids content and IC_50_ values of DPPH radicals scavenging.

As revealed in Figure 6, it was negative correlation (*R*^2^ = −0.812) and was not statistically significant (*p* > 0.05) on the correlation coefficients (*R*^2^) between total biflavonoid content and IC_50_ value of DPPH scavenging capacities illustrated. It showed that the experimental values exhibited a satisfactory mathematic model and the extracts with higher total biflavone yields usually had lower IC_50_ values, i.e., better antioxidant activity.

### 2.4. Comparison of Three Extraction Methods

As shown in Table 3, compared with soxhlet extraction on total biflavones yields and IC_50_ of DPPH radicals scavenging, ILs-MAE not only improved extraction efficiency and significantly reduced extraction time but also made the content of total biflavonoids increase nearly five times. Meanwhile, although extraction times of two methods was almost same, total biflavonoid yields were increased nearly two times compared to MAE. According to the comparison of three extracts IC_50_, ILs-MAE extract showed the best antioxidant activity. Through the contrast with two extraction methods, it was concluded that ILs-MAE has a great potential and was expected to be a rapid, efficient, and green method for total biflavonoids from *S. doederleinii*. The reproducibility of these three methods ranged from 97.71% to 99.34%, and the RSD was less than 3.00%.

### 2.5. Antioxidant of Total Biflavones

**Figure 7:**
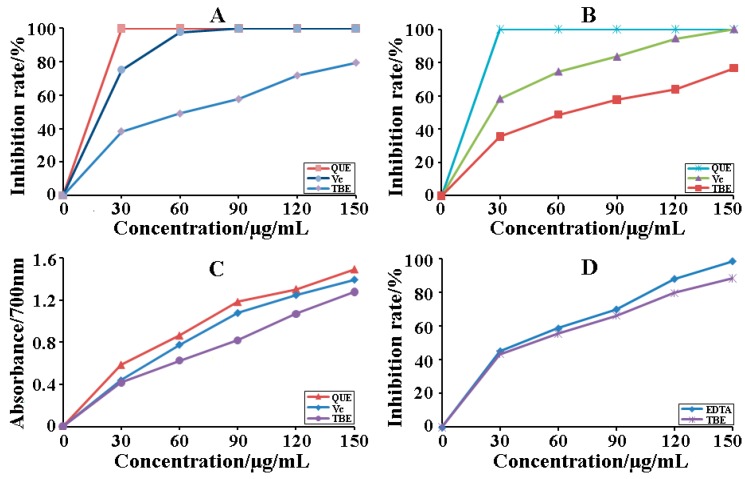
Antioxidant effects of TBE. (**A**) ABTS radical scavenging ability; (**B**) DPPH radical scavenging ability; (**C**) reducing power; (**D**) ferric chelation power.

Compared with the antioxidant activity of total biflavonoids extraction (TBE) with other antioxidants—i.e., positive controls (Vc, EDTA, quercetin)—four antioxidant approach were investigated including DPPH radicals scavenging, ABTS radicals scavenging, chelation of ferrous ions, and ferric reducing at different concentrations (30 μg/mL–150 μg/mL). As shown in Figure 7A, the results revealed that the scavenging effects of three samples on ABTS radical activities decreased successively in the following order: quercetin > Vc > TBE. In addition, the inhibition percentage of three samples was obviously added with the improvement of each sample concentration. It was concluded that TBE exhibited a better radical scavenging activity and the maximum ABTS inhibition percentage of TBE was 76.59% at 150 μg/mL.

The DPPH assay was also a common radical scavenging method of natural antioxidants. When considering the antioxidant effects of three samples, the results were seriously evaluated in Figure 7B. The results indicated that quercetin has the strongest radical scavenging capacity, and Vc, TBE follow suit. Moreover, the inhibition percentage of three samples was obviously added with the improvement of each sample concentration. It was concluded that TBE exhibited a better radical scavenging activity and the maximum DPPH inhibition percentage of TBE was 79.50% at 150 μg/mL. The results of analysis were almost consistent with those of DPPH radical scavenging mentioned above (Figure 7B).

Reducing capacity test was usually evaluated the antioxidant ability of natural products by the principle of Fe^2+^ reduction [38]. Figure 7C implied that all the samples exhibited similar activities at high concentration. When the concentration of three samples was constantly increased, the reducing power significantly increased. However, the antioxidative activity of TBE was lower than that of quercetin and Vc at different concentrations. Based on the results of the above-mentioned analysis combined related literatures, scholars thought the antioxidant capacity of the biflavone might depend on the number and position of the hydroxyl groups in its aromatic ring [39]. 

According to the methods adopted by Benzie and Strain [40], chelation power of ferrous principle was described as follows: iron ions were known to promote the conversion of less reactive species such as hydroxyl, peroxyl/alkoxyl radicals and the release of iron ion can accelerate oxidative damage. Figure 7D indicated that two samples showed similar activities at high concentration. Meanwhile, ferrous chelation capacity increased significantly following the improvement of two samples. In addition, the antioxidative activity of TBE was lower than that of EDTA at different concentrations. Our results were generally consistent with ferrous reducing capacity data mentioned above (Figure 7C).

### 2.6. Anticancer Activity

As showed in Table 4, the anticaner activity of TBE against A-549 cell line and 7721 cell line was 120.51 *±* 8.09 μg/mL and 131.74 *±* 6.31 μg/mL, respectively. However, the anticaner activity of TBE was lower than that of Cis (20.89 ± 6.31 μg/mL and 6.27 ± 1.53 μg/mL, respectively). It showed that TBE exhibited a certain anticaner activity. Biflavones, as main effective components of *S. doederleinii,* showed definite anti-tumor effect in the related literature. For instance, scholars considered that ginkgetin could activate caspase-3 as a cysteine protease that played an important role in the process of invasion and metastasis of malignant tumor, and attenuate the expression of survival genes such as Bcl-2, Bcl-x(L), PC-3, survivin and Cyclin D_1_ at protein and mRNA levels as a potent chemotherapeutic agent for prostate cancer treatment [41]. As another example, Lee [42] and coworkers separated amentoflavone from *Selaginella tamariscina* and found it could dose-dependently inhibit FASN that played main role by providing the nutrients needed for tumor growth and proliferation in breast cancer, and further inhibit MCF-7 cell line growth. Meanwhile, it could reduce fatty acid synthesis by reducing combination of acetyl-COA in human breast cancer cells *S*K-BR-3. The results indicated that biflavonoids had main contribution to anticancer activities of the extract from *S. doederleinii*

## 3. Experimental Section

### 3.1. Chemicals and Materials

The herb sample was collected at Guizhou Simianshan on 21/08/2015 and identified as *S. doederleinii* by vice-professor Zhang yu-jin from the department of Pharmacy in Zunyi medical college. A voucher specimen (No. 20150923) has been preserved in the Key Laboratory of Natural Pharmaceutical Chemistry, Zunyi Medical College. 2,2-diphenyl-1-picrylhydrazyl(DPPH), 2,2‘-azinobis-(3-ethylbenzthiazoline-6-sulphonate) (ABTS) and 3-(2-Pyridyl)-5,6-bis(4-sulfophenyl)-1,2,4-triazine disodium salt (Ferrozine), Vitamin C, Quercetin, ethylene diamine tetraacetic acid(EDTA), 3-(4,5-dimethyl-thiazolyl-2)-2,5-diphenyltetrazolium bromide (MTT) and cisplatin were purchased from Sigma Pure Chemical Industries (Berlin,Germany). 1-butyl-3 -methylimidazolium hexafluoroarsenate ((Bmim) (PF_6_)), 1-butyl-3-methylimidazolium bromine ((Bmim) (Br)), 1-butyl-3-methylimidazolium tetrafluoroborate ((Bmim) (BF_4_)), 1-butyl-3-methyli-midazolium acetate ((Bmim) (OAC)), 1-hexyl-3-methylimidazolium hexafluoroarsenate ((Hmim) (PF_6_)) and 1-octyl-3-methylimidazolium hexafluoroarsenate ((Bmim) (PF_6_)) were purchased from Chengjie Chem Ltd (ShangHai, China). RPMI-1640 culture was purchased from YuanLong Biotechnology Company (ShangHai, China). All other reagents were used as analytical grade and purchased from ChangZheng Chemical Company (Chengdu, China). Human cancer dupoumon cell strain (A549) and human hepatic carcinoma cell strain (7721) were purchased from Shanghai cell bank of Chinese Academy of Sciences.

### 3.2. Sample Treatment 

Dried *S. doederleinii* was powdered and then stored at −5 °C in a refrigerator. An accurate amount (10.0 g) of these powders was mixed with a defined amount of extraction solvent. the sample in reaction flask was irradiated for the pre-set microwave power, extraction temperature, and extraction time in NH-100 microwave extraction apparatus (Milkyway instrument company, Beijing, China), filtrated and centrifuged at 4000 rpm under 5 °C in SF-TDL-6A high speed centrifuge (Qiafeier analytical instrument company, Shanghai, China). After the supernatant was concentrated and dried under vacuum, total biflavone extraction (TBE) was obtained and stored at 5 °C until analysis. All the experiments were carried out in triplicate.

### 3.3. Determination of Total Biflavonoid Content

The determination method of total biflavonoids was subtly adjusted by aluminum chloride colorimetric assay [43]. In short, 0.6 mL of the extract was mixed with 1.6 mL methanol, 0.2 mL of 10% aluminum chloride, 0.2 mol·mL potassium acetate and 2.9 mL of distilled water. The mixture was reacted at room temperature for 40 min, and then the absorbance was measured at 415 nm with a TU-1800 UV-spectrophotometer (Puxi general instrument Co. Ltd., Beijing, China). The concentration of biflavonoids was calculated by plotting the standard quercetin calibration curve. Total biflavonoids content of the extract was expressed according to the following formula:

Yields of TBE (%) = (quercetin equivalents (mg)/dry extract (g)) × 100(3)

### 3.4. Experimental Design

#### 3.4.1. Single-Factor Experiments

To evaluate experiment effect on the biflavones of the extract from *S. doederleinii*, microwave power (100, 300, 500, 700, and 900 W), solid-liquid ratios (1:5, 1:10, 1:15, 1:20, and 1:25 g/mL), extraction time (l0, 20, 30, 40, 50, and 60 min) and ILs concentration(1.0, 1.5, 2.0, 2.5, 3.0 mmol/L), extraction temperature (30, 40, 50, 60, and 70 °C) were investigated as single factor variables (Table 5).

#### 3.4.2. Response Surface Methodology Experiments

Response surface method combined MAE-assisted technology was usually employed to optimize the craft parameter in the extraction of natural plant [44]. On the basis of single factor experiments, three factors affected significantly, i.e., microwave power (X_1_), extraction time (X_2_), and extraction temperature (X_3_), were selected as the independent variables. The effects of three independent variables on the content of total biflavonoids (Y_1_) and IC_50_ of DPPH radicals scavenging (Y_2_) were investigated with a BBD statistical model of RSM (see Table 6) to fit the second-order polynomial equations as follows:

Y = β_0_ + β_1_X_1_ + β_2_X_2_ + β_3_X_3_ + β_11_X_1_^2^ + β_22_X_2_^2^ + β_33_X_3_^2^ + β_12_X_1_X_2_ + β_13_X_1_X_3_ + β_23_X_2_X_3_ (4)

### 3.5. Comparison of Various Extracting Methods

#### 3.5.1. Microwave-Assisted Extraction

The powders (10.0 g) were placed in a reaction flask (250 mL) and mixed with 100 mL of 70% ethanol. Next, the mixture was irradiated with 460 W microwave power at 45 °C for 45 min in a microwave extraction apparatus, filtrated, and centrifuged at 4000 rpm under 5 °C. The supernatant was concentrated and dried under vacuum and stored at 4 °C until use. 

#### 3.5.2. Soxhlet Extraction

The powders (10.0 g) were kept on a Whatman filter paper. The samples were mixed well with 200 mL of 70% ethanol and extracted for 2 h at 95 °C in a Soxhlet extractor [45,46]. After extraction, the sample was taken out, filtrated, and centrifuged at 4000 rpm under 5 °C. The supernatant was concentrated, dried under vacuum, and stored at 4 °C until analysis.

### 3.6. Evaluation of Antioxidant Properties

#### 3.6.1. ABTS Radical-Scavenging Activity 

The determination method of ABTS radical scavenging ability was subtly improved by reference of Du [47]. 6 mmol/L ABTS solution was mixed well with 120 mmol/L potassium persulfate water solution (ultimate concentration) and left standing overnight in the dark at room temperature to generate ABTS^+^. TBE was dissolved with methyl alcohol and was diluted to different concentrations sample (30.0, 60.0, 90.0, 120.0, 150.0 μg/mL) 0.5 mL of each sample was mixed with 2.0 mL ABTS^+^ solution, left standing for 10 min at room temperature and determined the absorbance of the sample at 734 nm in a ultraviolet spectrophotometer. The scavenging rate can be calculated according to the following formula:

*I*(%) = ((A_b_ – A_s_)/Ab) × 100% (5)

where *I* was the inhibition percentage, A_b_ was the absorbance of the blank sample, and A_s_ was the absorbance of the test sample. Sample concentration providing 50% inhibition (IC_50_) was calculated by plotting the inhibition percentage against different concentrations sample. Ascorbic acid and quercetin (in the 30–150 μg/mL range) were used as positive controls. The entire test was run in triplicate and the average value was calculated.

#### 3.6.2. DPPH Radical-Scavenging Activity

Determination of antioxidant activity was performed using the method [48]. 0.7 mL of the extract with different concentrations was mingled with 2.2 mL DPPH ethanol solution (0.2 mmol/L) separately. The mixture was shaken hard, produced a reaction, and was left standing at room temperature in the dark for 60 min. The absorbance was measured at 517 nm in the ultraviolet spectrophotometer. IC_50_ was calculated by plotting the inhibition percentage against different concentrations sample. Ascorbic acid and quercetin (in the range of 30 μg/mL–150 μg/mL) were used as positive controls. 

#### 3.6.3. Evaluation for Reducing Power

The reducing power of the samples was measured using the method of Kim [49] with some modification. 1.2 mL of the sample with different concentrations were mingled with 1.0 mL phosphate buffer solution (PBS, 0.2 mol/L, pH 6.6) and 1.0 mL 1% potassium ferricyanide. The mixture was reacted for 20 min in 50 °C and quickly chilled. Afterwards, the mixture was mixed well with 1.0 mL 10% trichloroacetic acid and 5 mL distilled water, and then centrifuged at 3000 r/min for 10 min. 2.5 mL of the supernatant was mixed with 0.5 mL 0.1% ferric trichloride solution and 2 mL distilled water and left standing for 10 min. Absorbance (A) was measured at 700 nm in the ultraviolet spectrophotometer. Ascorbic acid and quercetin (in the range of 30 μg/mL–150 μg/mL) were used as reference compounds. 

#### 3.6.4. Evaluation for Chelation of Ferrous Ions

The chelation of ferrous ions is estimated using the method of Benzie and Strain [50]. 0.1 mL of each sample was added to a solution of 0.7 mL ferrous chloride (0.2 mmol/L). The mixture was diluted with 1.9 mL distilled water and reacted with 0.6 mL of ferrozine (5 mmol/L) at room temperature for 10 min. Absorbance was measured at 562 nm and then the IC_50_ of different samples was calculated. EDTA (in the range of 30 μg/mL–150 μg/mL) was used as reference compound.

### 3.7. Evaluation for Anticancer Activity

A549 and 7721 cancer cell lines were maintained in RPMI-1640 medium supplemented with 100 g/L heat-inactivated (56 °C) fetal bovine serum, 3 mM/L glutamine, 100 mg/mL streptomycin and 100 IU/mL penicillin, and the above system was adjusted to pH 7.2 with bicarbonate solution [51,52]. Cells were grown in a humidified atmosphere of 95% air/5% CO_2_ (*v/v*) at 37 °C. 20 μL of TBE with different concentrations (100, 200, 300, 400, and 500 μg/mL) were mingled with PBS, and then the mixture was added into each microwell and subcultured in mediums for 44 h. After that, the mixture was mixed with 50 μL MTT solution (1 mg/mL, dissolved by PBS) in each microwell, and then incubated for 4 h again. The supernatant in the microwell was carefully removed and separated from the culture medium. Afterwards, the mixture was mingled with 150 μL DMSO and shaken for 10 min to dissolve the methanol crystal fully. Absorbance of the sample was measured at 570 nm in the enzyme-linked immunosorbent assay tester (DG5033A, Huadong electronics Co. Ltd., Nanjing, China). IC_50_ values of the samples were calculated by plotting the inhibition percentage against different concentrations sample. Cisplatin (in the 100–500 μg/mL range) was used as a positive control. 

## 4. Conclusions

In order to develop a novel, efficient, and green extraction method, IL-MAE was successfully employed in the extraction process towards total biflavones and IC_50_ of DPPH radical scavenging for the first time based on single-factor experiments and response surface optimization in TBE from *S. doederleinii*. Optimal conditions were obtained as follows: ILs concentration, 2.0 mmol/L; solvent-material, 1:15 g/mL; extraction time, 40 min; temperature, 45 °C; microwave power, 460 W. Furthermore, the proposed IL-MAE was more efficient than MAE and Soxhlet extraction methods at a shorter time and lower temperature. Meanwhile, TBE exhibited better antioxidation by four assays, which included DPPH and ABTS radical scavenging, ferric reducing power, and ferric chelating ability, and certain antitumor activity against A549 cell line and 7721 cell line. The results showed that *S. doederleinii* was a good natural source of biflavones and IL-MAE was an efficient extraction method for biflavonoids from natural products. Therefore, this study of certain reference value for further development of the application of biflavonoids in food and medical domains.

## Figures and Tables

**Table 1:** ANOVA for the fitted quadratic polynomial model for total flavonoids content.

Source	Sum of Squares	df	*F*	*P*	*R^2^*	*R*^2^ *(Adj)*
Model	54.55	9	55.39	<0.0001	0.9862	0.9684
X_1_	2.76	1	25.23	0.0015		
X_2_	0.59	1	5.43	0.0526		
X_3_	10.58	1	96.69	<0.0001		
X_1_X_2_	2.250 × 10^−4^	1	2.056 × 10^−3^	0.0304		
X_1_X_3_	0.80	1	7.32	0.6743		
X_2_X_3_	0.021	1	0.19	0.0165		
X_1_^2^	13.05	1	119.29	<0.0001		
X_2_^2^	8.20	1	74.96	<0.0001		
X_3_^2^	14.42	1	131.80	<0.0001		
Residual	0.77	7				
Lack of Fit	0.65	3	7.71	0.0387		
Pure Error	0.11	4				
Cor Total	55.32	16				

**Table 2:** ANOVA for the fitted quadratic polynomial model for IC_50_ value of antioxidant activity.

Source	Sum of Squares	df	*F*	*P*	*R^2^*	*R*^2^ *(Adj)*
Model	1659.94	9	36.07	<0.0001	0.9879	0.9518
X_1_	13.49	1	2.64	0.1483		
X_2_	3.91	1	0.77	0.4107		
X_3_	243.49	1	47.62	0.0002		
X_1_X_2_	63.64	1	12.45	0.0096		
X_1_X_3_	88.03	1	17.22	0.0043		
X_2_X_3_	1.68	1	0.33	0.5848		
X_1_^2^	469.26	1	91.77	<0.0001		
X_2_^2^	509.31	1	99.60	<0.0001		
X_3_^2^	146.11	1	28.57	0.0011		
Residual	35.79	7				
Lack of fit	19.97	3	1.68	0.3070		
Pure Error	15.83	4				
Cor Total	1695.73	16				

**Table 3:** Comparison of different extract methods on total biflavonoids content and IC_50_ values.

Extraction Method	ILs-MAE	MAE	Soxhlet Extraction
Relative extraction rate ^a^ (mean ± SD, %)	100.00 ± 9.13	53.69 ± 1.49	29.51 ± 1.06
IC_50_ (mg/g)	56.17 ± 2.21	68.35 ± 1.77	81.09 ± 3.92
Extraction time (min)	40	45	120

^a^ Extraction rate is the actual content of biflavonoids observed, The maximum extraction rate of total biflavonoids was set as 100% and relative extraction rate of the sample that were extracted with MAE and Soxhlet method were calculated.

**Table 4:** IC_50_ values (μg /mL) of TBE for cytotoxicity test.

Treatment	A549 Cell Line	7721 Cell Line
TBE	120.51 ± 8.09	131.74 ± 6.31
Cis	20.89 ± 6.31	6.27 ± 1.53

**Table 5:** Level and code of factors chosen for experiment.

Level	Factors		
	Microwave Power (W)	Extract Time (min)	Extract Temper (°C)
−1	300	30	40
0	500	40	50
1	700	50	60

**Table 6:** The results of experiment design.

Run	X_1_ (Microwave Power/W)	X_2_ (Extraction Time/min)	X_3_ (Extraction Temper/°C)	Y_1_ (mg/g)	IC_50_ (μg/mL)
1	700	40	60	12.07	78.82
2	500	40	50	16.52	61.11
3	500	40	50	16.83	59.24
4	500	40	50	16.52	56.25
5	500	40	50	16.83	60.46
6	300	40	40	15.01	63.83
7	500	50	40	14.73	70.22
8	500	30	60	12.33	81.41
9	500	30	40	15.06	70.82
10	500	40	50	16.82	60.78
11	700	50	50	12.67	75.58
12	300	30	50	14.44	78.69
13	700	30	50	13.28	87.05
14	300	50	50	13.80	83.17
15	300	40	60	12.38	83.40
16	700	40	40	12.91	78.02
17	500	50	60	11.72	83.39
